# Ice-Binding Protein from *Shewanella frigidimarinas* Inhibits Ice Crystal Growth in Highly Alkaline Solutions

**DOI:** 10.3390/polym11020299

**Published:** 2019-02-11

**Authors:** Elizabeth A. Delesky, Shane D. Frazier, Jaqueline D. Wallat, Kendra L. Bannister, Chelsea M. Heveran, Wil V. Srubar

**Affiliations:** 1 Materials Science and Engineering Program, University of Colorado Boulder, Boulder, CO 80309, USA; elizabeth.delesky@colorado.edu (E.A.D.); shane.frazier@colorado.edu (S.D.F.); 2 Department of Civil, Environmental, and Architectural Engineering, University of Colorado Boulder; Boulder, CO 80309, USA; jaqueline.wallat@colorado.edu (J.D.W.); chelsea.heveran@colorado.edu (C.M.H.); 3 Department of Chemical and Biological Engineering, University of Colorado Boulder; Boulder, CO 80309, USA; kendra.bannister@colorado.edu

**Keywords:** ice-binding protein, ice recrystallization inhibition, alkalinity, ionic strength

## Abstract

The ability of a natural ice-binding protein from *Shewanella frigidimarina* (SfIBP) to inhibit ice crystal growth in highly alkaline solutions with increasing pH and ionic strength was investigated in this work. The purity of isolated SfIBP was first confirmed via sodium dodecyl sulfate polyacrylamide gel electrophoresis (SDS-PAGE) and size-exclusion chromatography with an ultraviolet detector (SEC-UV). Protein stability was evaluated in the alkaline solutions using circular dichroism spectroscopy, SEC-UV, and SDS-PAGE. SfIBP ice recrystallization inhibition (IRI) activity, a measure of ice crystal growth inhibition, was assessed using a modified splat assay. Statistical analysis of results substantiated that, despite partial denaturation and misfolding, SfIBP limited ice crystal growth in alkaline solutions (pH ≤ 12.7) with ionic strength *I* ≤ 0.05 mol/L, but did not exhibit IRI activity in alkaline solutions where pH ≥ 13.2 and *I* ≥ 0.16 mol/L. IRI activity of SfIBP in solutions with pH ≤ 12.7 and *I* ≤ 0.05 mol/L demonstrated up to ≈ 66% reduction in ice crystal size compared to neat solutions.

## 1. Introduction

### 1.1. Ice-Binding Proteins

Ice-binding proteins (IBPs) are a robust series of proteins found in a multitude of freeze-avoidant and freeze-tolerant organisms, including fish, fungi, plants, and bacteria, that are capable of surviving sub-zero temperatures by inhibiting ice crystal growth and controlling ice crystal morphology [1,2,3,4,5]. Some freeze-tolerant organisms produce IBPs that prevent the coalescence of small, nucleated ice crystals into larger, more destructive crystals through a mechanism known as ice recrystallization inhibition (IRI) [6]. The growth of large ice crystals at the expense of smaller crystals is thermodynamically preferred to minimize interfacial energy at the grain boundaries [7,8]. Mechanistically, IBPs function in a non-colligative manner for IRI through lattice matching of the protein ice-binding face with the crystal lattice of ice, which induces a high local curvature and increases the energy required for further crystal growth [5,9,10,11,12].

Previous cryogenic research indicates that IBPs may offer a new, biomimetic alternative to conventional frost-prevention strategies for biological materials [5,13] and, by extension, antifreeze applications in a host of other commercial industries (e.g., aerospace, infrastructure). Previous research indicates that low concentrations of IBPs can be used to cryopreserve microorganisms, such as microalgae used to produce insulin [14], to improve the viability of rat kidneys post-thaw over conventional agents in media [15], and to ameliorate follicular integrity of vitrified-warmed mouse ovaries [16]. Additionally, the efficacy of IBPs to reduce hemolysis of red blood cells upon thawing has previously been investigated: An IBP from the genus *Leucosporidium* significantly reduced hemolysis at concentrations of 0.4–0.8 mg/mL [17], and three IBPS (AFPI, AFPII, and AFPIII) were shown to reduce hemolysis by 75% compared to controls [18]. The effects of IBPs on cryopreservation have been found to depend on IBP type and concentration, the preservation protocol, and biological material [19]. As an emerging biotechnology, IBPs have the potential to extend beyond biological applications to meet frost-prevention needs of other industries in aerospace (e.g., cryogenic fluids), civil engineering (e.g., frost-resistant pavements), and energy infrastructure (e.g., anti-icing coatings). While IBPs offer a promising biological solution for these ice-prevention applications, proteins are well known to restructure (e.g., unfold, refold, denature, aggregate, degrade) in non-native environments [20]. Changes in pH and ionic concentration may affect IRI activity of IBPs and limit their applicability as a biotechnological frost-resistance solution in novel applications with more aggressive chemical environments. 

IBPs have been shown to exhibit control of ice structures at nanomolar (nM) concentrations of IBPs in solution [21,22], and a few studies have indicated that IBPs may perform similarly in ionic solutions [23,24]. While IRI was not reported, Kristainsen et al. [23] found that antifreeze activity as measured by thermal hysteresis using nanoliter osmometry for *Rhagium inquisitor* IBP was improved six-fold in 0.8 M monovalent ionic solutions of tri-sodium citrate, sodium chloride (NaCl), and sodium iodide. Leiter et al. [24] studied the performance of Type III fish antifreeze protein in low concentrations of NaCl (i.e., 20–30 mM) and found a marginal increase in IRI activity compared to neat solutions. Leiter et al. also investigated the effect of 0.1 M NaOH (pH 11) on the IRI activity of Type III fish antifreeze protein and found that the elevated pH did not affect IRI activity [24]. Taken together, these studies indicate the potential for IBPs to maintain IRI activity in non-native ionic environments. 

### 1.2. Scope of Work

The purpose of this work was to investigate the ability of an ice-binding protein from the bacterium *Shewanella frigidimarinas* (SfIBP) to control the size and inhibit the growth of ice crystals in highly alkaline solutions (pH > 12) with increasing ionic strength. First, the structural stability of SfIBP was investigated using circular dichroism (CD) spectroscopy. Second, SfIBP stability, aggregation, and degradation were analyzed with two protein size-analysis techniques, sodium dodecyl sulfate polyacrylamide gel electrophoresis (SDS-PAGE) and size-exclusion chromatography with an ultraviolet detector (SEC-UV). Finally, SfIBP IRI activity was investigated using a modified splat assay and compared to controls of neat solutions. Similar to precedent research [25,26,27,28], IRI activity was determined through direct measurement of the mean size of ice crystals that formed in the alkaline solutions that contained SfIBP after incubation in freezing (−4 °C) conditions compared to neat alkaline solutions.

## 2. Materials and Methods 

### 2.1. Materials

Calcium hydroxide (Ca(OH)_2_), potassium hydroxide (KOH), sodium hydroxide (NaOH), calcium sulfate (CaSO_4_), 2-mercaptoethanol, and bovine serum albumin (BSA) were purchased from Sigma Aldrich without further purification. Tris(hydroxymethyl)aminomethane buffer (Tris) was purchased from Fisher Bioreagents without further purification. *Shewanella frigidimarina* IBP isoform 1 (SfIBP) at a concentration of 4 mg/mL in solution was obtained from Dr. Peter Davies at Queen’s University in Kingston, Ontario, Canada [22] and was reconstituted using a centrifugal filter into 20 mM Tris solution. SfIBP concentration was verified at 4.4 mg/mL against BSA using UV-Vis. 

Hydroxide salts were used to create alkaline solutions of increasing pH in ~0.5 pH increments from pH ~12.5 to 14.0. Formulations were adapted from studies performed by Ghods et al. [29], and the supernatant decanted. The cation concentrations in the supernatant were verified using inductively coupled plasma mass spectrometry (ICP-MS), and hydroxide ion concentrations were determined from solution pH (Table 1). Tris buffer was included in all solutions to account for protein addition. Total ionic strength (*I*) for each solution was calculated according to Equation (1):

(1) $$I=\frac{1}{2}\sum Z^{2}C,$$

where *Z* is the valence of the ion and *C* is the ion concentration. As Tris has a pK_a_ of 8.1, solutions with pH > 12 were above Tris’s buffer capacity. Therefore, Tris was determined to have dissociated completely to its conjugate base (deprotonated, uncharged) and conjugate acid (H^+^), as per the Henderson-Hasselbach equation. Since Tris was in its deprotonated form, it was not included in the ionic strength calculations for solutions with pH > 12. The conjugate acid was expected to neutralize through combination with hydroxide ions present in alkaline solutions to form water, which would have been reflected in measured solution pH used to determine hydroxide concentration. Samples for IRI characterization without or with 0.125 mg/mL SfIBP were prepared using stock solutions from Table 1. While SfIBP has been shown to exhibit activity in protein buffer conditions at concentrations as low as 50 nM (0.00125 mg/mL) [22], lower SfIBP concentrations tested did not exhibit inhibition in this study (data not shown) due to the ionic and alkaline nature of the solutions. Therefore, a concentration of 0.125 mg/mL was selected in this study to evaluate ice crystal nucleation and growth inhibition. 

### 2.2. Experimental Methods

#### 2.2.1. CD Spectroscopy

SfIBP structure and stability were analyzed in two solutions (Table 1), namely 1/2 Tris and A + 1/2 Tris, via circular dichroism (CD) spectroscopy in the far UV range (190–260 nm) using a modular Applied Photophysics Chirascan Plus CD and Fluorescence Spectrometer at ambient temperature with 0.5 nm steps and 0.5 sec/step at a 0.5 mm path length. SfIBP was loaded at 0.5 mg/mL for CD analysis for improved protein signal. SfIBP could not be analyzed in all solutions, given the confluence of increasing alkalinity and ionic strength and the signal detection limits of the instrument. Secondary structure composition (% helix, sheet, turns, etc.) was measured from the peptide bond region (<240 nm) [30] using BeStSel software [31]. SfIBP was allowed to incubate in solution for at least 24 hours to ensure equilibrium folding states [32], as it was expected that the alkalinity and high ion concentrations would induce protein misfolding. All CD spectra were averaged over five runs on the same sample and the solution baseline was removed from the spectra. Curves were smoothed to remove noise using the Savitzky-Golay filter method in OriginPro 2016 using 5 points per window with a polynomial order of 2. 

#### 2.2.2. SEC-UV

SfIBP stability, aggregation, and degradation were analyzed using size-exclusion chromatography (SEC) equipped with an ultraviolet (UV) detector monitoring a wavelength of 220 nm. SEC was performed on an Agilent 1100 Series LC system with a UV detector and a Tosoh TSKgel G3000SWxl size exclusion column. For all experiments, the mobile phase was 100 mM potassium phosphate buffer (pH 7.4) at a flow rate of 0.4 mL/min. Solutions from Table 1 were analyzed without and with a SfIBP concentration of 0.4 mg/mL for improved signal. For each injection, 50 µL of sample were analyzed, resulting in a final SfIBP content of 20 µg. Data were processed using Astra software 7.1.2 and plotted using GraphPad Prism software 7.04.

#### 2.2.3. SDS-PAGE 

Sodium-dodecyl sulfate polyacrylamide gel electrophoresis (SDS-PAGE) was performed on SfIBP loaded into solutions from Table 1 at a concentration of 1 mg/mL to ensure visible bands in the gel. SfIBP samples were denatured prior to SDS-PAGE via the additional of 2-mercaptoethanol and subsequent heating at 95 °C for 5 minutes. Samples were loaded onto a 4–20% denaturing TGX gel from Bio-Rad (1.0 mm × 12 well; 35 min, 200 V, 1X Tris-Glycine-SDS PAGE running buffer, pH 8.8) and compared to a 10–250 kDa protein ladder (New England BioLabs) for estimation of molecular weight. The protein content within the gel was stained with Coomassie SimplyBlue SafeStain (Invitrogen) according to manufacturer specifications. 

#### 2.2.4. IRI Activity

A splat ice recrystallization assay was adapted from Knight et al. [26]. Solutions from Table 1 were tested neat or with a 0.125 mg/mL loading of SfIBP. A 10–20 µL droplet of solution was dispensed from 1.7 m through a PVC pipe onto a microscope slide on top of an aluminum block chilled with dry ice to obtain a monolayer of ice crystals. The slide was then transferred to an Otago nanoliter osmometer sample stage and annealed at −4 °C. The temperature was monitored using a bead-type thermocouple. Images were collected immediately after the splat was performed (t_0_) and again at 30 minutes (t_30_) to observe ice recrystallization. Images were obtained using a Zeiss Axio Imager M2m microscope with an EC Epiplan 5x/0.13 BD M27 objective and crossed polarizers, equipped with an Axiocam 506 color camera on a 1” 1.0x 60N C-mount adapter. ZENCore 2.4 image processing was used to measure individual grain sizes along the major axis. Data were taken from images from 2–3 different splat samples and used to determine an average grain size (*n* = 150) at *t* = 30 min. 

#### 2.2.5. Statistical Analyses

Grain sizes were first averaged for each of the replicate images. The effect of solution (i.e., different ionic strength and pH per Table 1) and inclusion of protein on mean grain size, as well as the interaction between these factors, was then tested using two-factor ANOVA. Model assumptions of residual normality and homoscedasticity were satisfied. For main effects, significance was set *a priori* to *p* < 0.05. Simple effects (i.e., the effect of protein on grain size for a particular solution) were assessed with the Fisher Least Significant Difference test using a Bonferroni correction to account for family-wise error (critical *α* = 0.05/3 = 0.0167). To determine if ionic strength and pH influenced mean grain size, these two solution chemistry metrics were first tested for intercorrelation using Pearson product-moment correlation. Since ionic strength and pH were found to be intercorrelated, no conclusions about the relative influence of pH versus ionic strength on mean grain size could be made. All statistical analyses were performed with Minitab (v18).

## 3. Results

### 3.1. CD Spectroscopy

**Figure 1:**
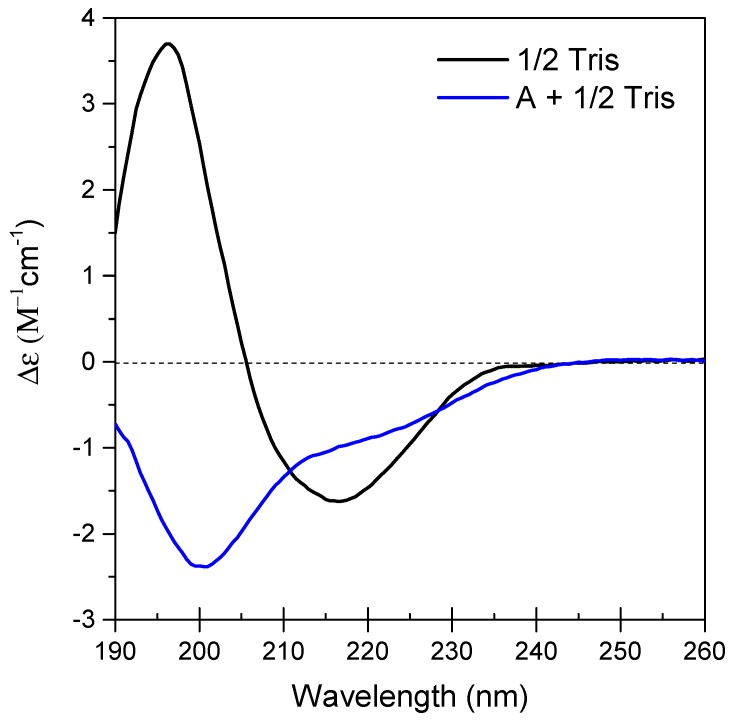
CD spectra showing SfIBP secondary structure at 0.5 mg/mL in 1/2 Tris (—) and A + 1/2 Tris (—). Spectra for B, C, and D were not attainable due to oversaturation of CD absorption.

Results from protein stability and secondary structure determination using CD are shown in Figure 1. As expected, SfIBP exhibited an initially well-folded secondary structure in 1/2 Tris (Figure 1) that matches previously reported spectra for SfIBP [22]. As anticipated, proteins incubated in solution A + 1/2 Tris at ambient conditions exhibited partial misfolding (Figure 1). 

Since CD uses plane polarized light absorbance to analyze protein composition, highly ionic solutions can saturate the absorbance detectors in CD. Therefore, ion concentrations in solution were increased (A→D) to find the maximum ionic strength that did not saturate the CD detector, which corresponded to solution A with Tris concentration reduced to 10 mM (A + 1/2 Tris). CD spectra could not be obtained for solutions B, C, or D, as the ion concentration of the solutions saturated CD absorption, preventing detection of SfIBP in the peptide bond region.

An analysis of secondary structure using CD data of SfIBP in 1/2 Tris and its associated changes when placed in A + 1/2 Tris is presented in Table 2. Data analysis using BeStSel software parsed secondary structure of SfIBP into 8 categories: Regular α-helix, distorted α-helix, left β-helix, relaxed β-helix, right β-helix, parallel β-strand, turn, and other (disordered). Table 2 lists the relative percentages of each secondary structure identified for SfIBP in 1/2 Tris and the relative changes to those structures when placed in A + 1/2 Tris. Values are expressed as a positive or negative percent. 

### 3.2. SEC-UV and SDS-PAGE

**Figure 2:**
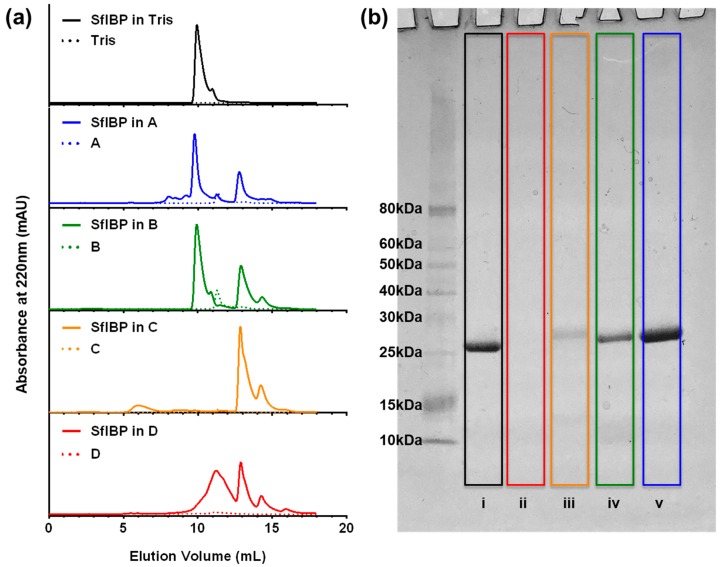
(**a**) SEC-UV absorbance at 220 nm as a function of elution volume for SfIBP in Tris and alkaline solutions with increasing ionic strength. (**b**) SDS-PAGE for SfIBP. Left to right, SfIBP in: (i) Tris; (ii) solution D; (iii) solution C; (iv) solution B; (v) solution A.

SEC-UV and SDS-PAGE were used to analyze protein stability, aggregation, and degradation in the alkaline solutions investigated herein. SEC-UV data are presented in Figure 2a. The chromatograms show UV absorbance signals from SfIBP as a function of elution volume from the column. SfIBP in Tris exhibited a singular dominant peak around 10 mL, similar to the singular peak exhibited by Vance et al. [22]. The SfIBP signal in alkaline solutions with *I* ≤ 0.05 mol/L (solutions A and B) had a shape and elution volume similar to SfIBP in Tris (Figure 2a). Additional UV signals appeared for SfIBP in all alkaline solutions at greater elution volumes with lower UV absorbance than the main protein elution at ~10 mL. In solution A, SfIBP exhibited a peak at the 10 mL mark, similar to Tris, along with other prominent peaks past 12.5 mL. Similarly, for solution B, SfIBP exhibited one prominent peak that matches SfIBP in Tris at 10 mL, but residual peaks were evident after 12.5 mL. SfIBP in solution C exhibited peaks after 12.5 mL—as do all other solutions—but manifests a peak around 6 mL and distinctly lacks a peak at 10 mL. SfIBP in solution D exhibited absorbance peaks that are shifted to greater elution volumes than SfIBP in Tris and exhibited peak broadening.

The SDS-PAGE results are presented in Figure 2b. The results for SfIBP in Tris and alkaline solutions are depicted with decreasing ionic concentrations (solution D→A). SfIBP in Tris exhibited a large, single band ≈ 25 kDa, matching the molecular weight of SfIBP as previously reported by Vance et al. [22]. SfIBP incubated in solution D did not demonstrate any obvious bands for SDS-PAGE. However, in solutions where *I* ≤ 0.16 mol/L (i.e., solutions A, B, C), bands corresponding to intact SfIBP protein ≈ 25 kDa are evident. SfIBP exhibited faint bands in lower molecular weight regions (≈10 kDa) for all alkaline solutions except for solution D.

### 3.3. IRI Activity

**Figure 3:**
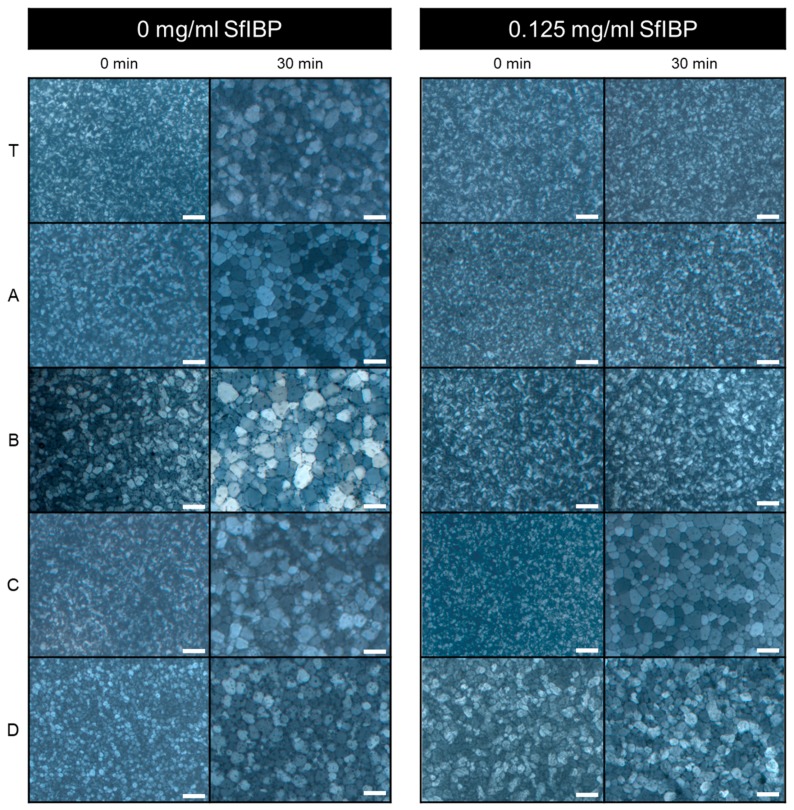
IRI activity of SfIBP in Tris buffer (T) and alkaline solutions (**A**–**D**) at 0 and 30 min. Scale bar = 100 µm.

**Figure 4:**
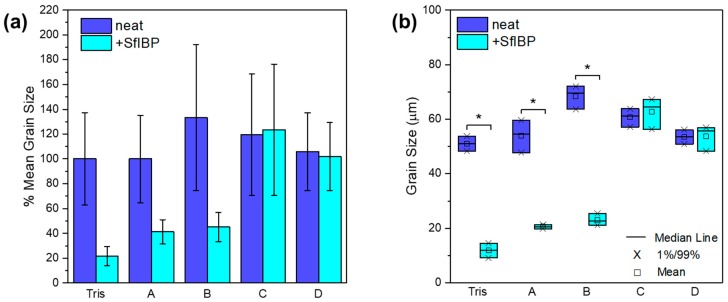
IRI activity of samples from Table 1 after incubation at −4 °C (*t* = 30 min) without SfIBP (■) and with SfIBP (■). (**a**) Data represented as % Mean Grain Size of ice crystals relative to neat Tris. Error bars equal ± one standard deviation. (**b**) Mean grain size is affected by solution and inclusion of protein. Asterisks indicate statistically significant differences in average grain size due to the addition of SfiBP (*p* < 0.001).

IRI activities of all solutions listed in Table 1 without (0 mg/mL) and with (0.125 mg/mL) SfIBP protein are demonstrated in Figure 3. The average size of ice crystallites formed in each solution after incubation at −4 °C is summarized in Table 3. The average percent difference of mean ice crystal grain size relative to neat Tris after incubation without and with SfIBP is demonstrated in Figure 4a, and statistical relevance of results as determined by ANOVA is shown in Figure 4b.

As expected, all solutions without SfIBP exhibited ice nucleation and growth upon incubation at sub-freezing temperatures (−4 °C). Ice crystallites with an average grain size of 51±19 µm formed in Tris without SfIBP, which were comparable in size to ice crystals formed in all alkaline solutions without SfIBP (Table 3).

SfIBP exhibited IRI activity in both Tris, as expected, and alkaline solutions (pH > 12) with *I* ≤ 0.05 mol/L, (i.e., solutions A and B). ANOVA determined that (i) solution, (ii) inclusion of protein, and (iii) the interaction between solution and protein all significantly affected mean grain size at *t* = 30 min. Simple effects testing revealed that for solutions A (−61.9%, *p* < 0.001), B (−66.3%, *p* < 0.001), and Tris (−76.9%, *p* < 0.001), including protein significantly reduced grain size (Figure 4b). SfIBP samples in Tris exhibited IRI activity, as evidenced by no noticeable ice growth beyond ice nucleation (Figure 3). When included in solution A, B, or Tris, SfIBP inhibited the growth of ice crystals (*p* < 0.0167) (Figure 4b). As expected, however, the inhibition was less than SfIBP in pure Tris. The IRI activity of SfIBP was similar in both solutions A and B, which had comparable ionic strengths of 0.03 mol/L and 0.05 mol/L, respectively. As summarized in Table 3, SfIBP in solutions C and D exhibited no IRI activity, where *I* ≥ 0.16 mol/L, as evidenced by final grain sizes that were not statistically different than their neat solutions (Figure 4b). The range of ionic strength in which SfIBP lost its ability to mitigate ice growth was 0.05 < I < 0.16 mol/L, and the range of pH for the loss of SfIBP function was 12.7 < pH < 13.2. Notably, in solutions where SfIBP exhibited ice growth inhibition (i.e., Tris, A, B), the distribution of crystal size was narrowed, as indicated by the smaller error bars.

## 4. Discussion

The structure and activity of the ice-binding protein SfIBP was investigated in solutions with high alkalinity and increasing ionic strength. The reduction in average ice crystal size in solutions A and B with SfIBP was statistically significant compared to neat solutions. However, in more alkaline solutions C (pH = 13.2) and D (pH = 13.9) with higher ion content (0.16 mol/L and 0.69 mol/L, respectively), the protein stability and IRI efficacy decreased, which affected ice recrystallization inhibition.

Despite misfolding and partial degradation, SfIBP exhibited secondary structure and ice-inhibiting functionality in alkaline solutions (pH = 12.4 to 12.7) with ionic strength *I* ≤ 0.05 mol/L. SfIBP degraded in solution D, as indicated by the lack of gel stain in SDS-PAGE (Figure 2b). Protein degradation is verified by the shifted peaks in SEC-UV (Figure 2a) to elution volumes greater than 12.5 mL in solution D. At *I* ≤ 0.16 mol/L, bands in SDS-PAGE at ≈ 25 kDa mirror SfIBP in Tris, indicating that SfIBP likely retained some of its structure in these solutions. SDS-PAGE verifies that SfIBP did not aggregate in any of the tested solutions due to the lack of molecular weight bands above 25 kDa. The peak broadening observed in SEC-UV absorbance for SfIBP in solution D (Figure 2a) corroborates the SDS-PAGE to indicate that protein secondary or tertiary structure was disrupted, likely due to changes in the proteins native charge, yielding chromatogram traces with peaks at larger volumes than the native protein due to ionic interactions with the column [33,34]. To this same end, the high alkalinity and ionic strength of the solutions likely facilitated covalent bond cleavage of the protein along the backbone through base-mediated hydrolysis [35,36], as indicated by faint bands in SDS-PAGE for SfIBP in solutions A, B, and C in the ≈ 10 kDa molecular weight region, and the lack of bands for solution D (Figure 2b).

According to SDS-PAGE and SEC-UV, SfIBP exhibited some protein degradation in all solutions. Sample eluting at volumes of 12.5 mL and greater indicate that the high alkalinity and ion content of the solutions likely caused some degree of protein restructuring and degradation [36,37]. At ionic strengths *I* ≤ 0.05 mol/L, chromatograms retained traces with absorbance peak shapes similar to SfIBP in Tris, along with evidence of some degradation. SfIBP in solutions A and B both exhibited UV absorbance peaks at 10 mL, similar to that of SfIBP in Tris, indicating retention of some protein. However, solutions C and D did not exhibit UV absorbance peaks at 10 mL, indicating degradation and, hence, a lack of protein structure that could exhibit IRI activity. The peak at 6 mL in solution C is decidedly an artifact due to the proportionally small signal, verified by the lack of higher molecular weight bands in SDS-PAGE.

Protein misfolding does not necessarily equate to loss of ice-binding functionality [20], as some ice-binding protein faces may still have been exposed to the solution through the process of refolding, retaining—albeit reducing—IRI activity. Secondary structure analysis (Table 2) quantitatively approximates residual protein structure. The analysis of SfIBP structure following CD experiments indicated that in solution A + 1/2 Tris the amount of unstructured protein increased by 5.2%, although the overall change in protein structure (taken as the difference between each type of fold), was altered approximately 37.7%. The current hypothesis for SfIBP IRI activity is closely related to β-fold content in the DUF3494 domain (domain of undefined function) [22]. The β-fold content in SfIBP secondary structure (left β-helix, relaxed β-helix, right β-helix, parallel β-strand) changed overall by ~ 23% when in A + 1/2 Tris compared to 1/2 Tris and is hypothesized to be responsible for the change in IRI activity. Based on the changes observed in CD spectra (Figure 1) and peak broadening in SEC-UV (Figure 2b), it can be deduced that, while SfIBP is misfolding, it retains some ice-binding functionality in solutions with high alkalinity (pH 12.4 to 12.7), as seen via IRI activity that is comparable to SfIBP in Tris (Figure 3 and Figure 4a). 

Due to the high alkalinity and ionic nature of the solutions (Table 1), it was expected that the secondary structure of SfIBP would become disordered, leading to disruption of protein tertiary structure that would affect its ice-binding capabilities [20]. The induced conformational changes and refolding are likely due to the disruption of hydrogen bonds necessary for proper protein folding in the native state [32]. It is hypothesized that SfIBP is refolding in response to the alkalinity of solutions, with either the ionic strength, the high pH, or a combination of both ionic strength and pH acting as a denaturant. 

Tris had a pH and ionic strength that promoted expected SfIBP conformation and function (Table 1, Figure 1 and Figure 3) and corresponded to the slightly basic solution pH, as verified in Vance et al. [22]. As Tris and solution A had comparable ionic strengths (*I* = 0.01 and *I* = 0.03 mol/L, respectively), the elevated pH of solution A likely caused SfIBP to misfold (Table 2). Despite the change in structure, however, SfIBP in solution A expressed a strong band at ≈ 25 kDa for SDS-PAGE (Figure 2b), a prominent absorbance peak at 10 mL for SEC-UV (Figure 2a), and IRI activity (Figure 3), indicating that SfIBP still maintained structure and functionality at elevated pH.

SfIBP in solution C did not evince IRI activity (Figure 3) and, as expected, showed clear signs of degradation, as seen in the lack of elution peak at 10 mL in SEC-UV (Figure 2a) and diminished intensity of the SDS-PAGE band at ≈ 25 kDa (Figure 2b). Solution D had the highest ionic strength (*I* = 0.69 mol/L) and highest alkalinity (pH = 13.9) of all tested solutions. Solution D was the only solution where SfIBP did not exhibit a band at ≈ 25 kDa in the SDS-PAGE gel (Figure 2b) and had a broadened peak in SEC-UV (Figure 2a), likely due to the extremely ionic and alkaline environment. 

While the folded structure of SfIBP in solutions with higher ionic content (e.g., solutions B, C, and D) cannot be determined directly through CD, it is hypothesized that further denaturation and decomposition occurred that prevented inhibition of ice crystal growth, as determined by the lack of IRI behavior of SfIBP in solutions C and D (Figure 3). The mean ice crystal grain size SfIBP in solutions C and D (63 ± 27 µm and 52 ± 14 µm, respectively) are similar in size to ice grains of solutions C and D without SfIBP (61 ± 25 µm and 54 ± 16 µm), indicating that the ice-binding face of the protein was no longer interacting with ice crystals nucleating and recrystallizing in solution (Table 3). However, given the retention of some protein structure in solution A + 1/2 Tris (Table 2), it was expected that SfIBP would still exhibit some inhibition of ice crystal growth, as substantiated in Figure 3, which shows that SfIBP in solutions A and B exhibit IRI activity. SfIBP in solutions A and B exhibited grain sizes of 21 ± 5 µm and 23 ± 6 µm, respectively, indicating the likelihood of protein interaction with ice crystals despite protein misfolding. While IRI activity was not as potent as in Tris, SfIBP effectively reduced average grain sizes in solutions A and B by ≈ 59% and ≈ 66%, respectively, compared to neat solutions (Figure 4a).

SfIBP exhibits IRI activity in alkaline solutions (pH > 12) with ionic strength *I* ≤ 0.05 mol/L, indicating that SfIBP (and other IBPs) could be effective at mitigating frost-induced damage in applications that necessitate activity in non-native chemical environments. It is clear from these data in Table 3 that solutions that exhibit IRI activity (i.e., SfIBP in Tris, A, and B) also demonstrate much narrower crystal size distributions, indicating that SfIBP not only inhibits ice crystal coalescence in these solutions, but dictates its final size in equilibrium. 

While SfIBP exhibits a potential to reduce frost-induced damage in select highly alkaline environments, other IBPs and new classes of biomimetic polymeric materials may prove more effective. Certain IBPs are well known to contain structures with a high density of stabilizing di-sulfide bonds, such as IBPs from *Tenebrio molitor* [38], which offer the potential of maintaining increased tertiary structure, and, thus, performance, in extremely ionic and alkaline environments. Other IBPs, such as those from *Marinomonas primoryensis* [39], require divalent calcium for proper folding and may be stabilized by calcium-rich environments with higher ionic strengths to maintain activity. In addition to proteins, polymer architectures that mimic the ice-binding functionality of IBPs offer a unique avenue for mitigating and controlling ice nucleation and growth, as they may not only be more cost-effective, but also able to inhibit ice crystal recrystallization in solutions of higher alkalinity without relying on tertiary structure or reduced ionic strength to exhibit IRI activity [40,41,42]. 

## 5. Conclusions

This study evaluated the potential of an ice-binding protein (IBP) from *Shewanella frigidimarinas* (SfIBP) to inhibit and control ice crystal nucleation and growth in highly alkaline solutions of increasing pH and ionic strength. While the folded structure of SfIBP in media with ionic strength *I* > 0.03 mol/L could not be determined directly through CD, based on evidence from SEC-UV and SDS-PAGE, it is assumed that a greater extent of denaturation and degradation occurred at higher ionic concentrations (*I* ≥ 0.16 mol/L) that prevented the inhibition of ice crystal growth, as determined by the lack of IRI behavior. Despite protein misfolding, data indicate that SfIBP exhibits ice recrystallization inhibition (IRI) activity in solutions with high alkalinity (pH = 12.4 to 12.7) and low ionic strength (*I* ≤ 0.05 mol/L) (≈ 66% reduction in ice crystal size compared to neat solutions). In conclusion, these results suggest that SfIBP (and other IBPs and their biomimetic synthetic replicates) could be effective at mitigating frost-induced damage in applications with chemically extreme non-native environments. 

## Figures and Tables

**Table 1:** Chemistry of alkaline solutions obtained via ICP-MS.

Solution	*I* (mol/L)	pH	OH (mM)	Tris (mM)	Ca (mM)	Na (mM)	K (mM)	S (mM)	Al (mM)	Mg (mM)	Si (mM)
A	0.03	12.4 ± 0.1	26.71	20	0.003	6.08	9.85	5.02	-	0.001	0.005
A + 1/2 Tris*	0.03	12.4 ± 0.1	23.62	10	0.003	6.08	9.85	5.02	-	0.001	0.005
B	0.05	12.7 ± 0.1	48.60	20	0.003	9.70	15.5	8.08	-	0.001	0.005
C	0.16	13.2 ± 0.1	168.5	20	0.007	23.9	37.3	20.6	0.001	0.002	0.005
D	0.69	13.9 ± 0.2	857.7	20	0.761	90.5	132.0	76.4	0.001	-	0.008
Tris	0.01	8.90 ± 0.1	0.008	20	-	-	-	-	-	-	-
1/2 Tris *	0.005	8.40 ± 0.2	0.003	10	-	-	-	-	-	-	-

* Indicates samples that were used for SfIBP circular dichroism (CD) characterization.

**Table 2:** SfIBP CD structure analysis in 1/2 Tris and A + 1/2 Tris.

Secondary Structure	1/2 Tris	A + 1/2 Tris	Difference
Regular α-helix	3.8%	2.1%	−1.7%
Distorted α-helix	5.1%	2.3%	−2.8%
Left β-helix	7.5%	1.0%	−6.5%
Relaxed β-helix	18.3%	10.4%	−7.9%
Right β-helix	13.2%	21.1%	+7.9%
Parallel β-strand	0.1%	0.8%	+0.7%
Turn	10.3%	15.3%	+5.0%
Other	41.7%	46.9%	+5.2%

**Table 3:** Average ice crystallite size of frozen solutions after incubation at −4 °C (*t* = 30 min).

Solution	SfIBP Loading (mg/mL)	Mean Crystal Size (µm)	% Change in Mean Crystal Size
Tris	0	51 ± 19	-
Tris	0.125	11 ± 4	−78%
A	0	51 ± 18	-
A	0.125	21 ± 5	−59%
B	0	68 ± 30	-
B	0.125	23 ± 6	−66%
C	0	61 ± 25	-
C	0.125	63 ± 27	+3%
D	0	54 ± 16	-
D	0.125	52 ± 14	+4%
